# A Case of Haemorrhagic Emphysematous Gastritis

**DOI:** 10.7759/cureus.66084

**Published:** 2024-08-03

**Authors:** Hussein Mansour, Jawad Ali, Anupama Swamy, Anthony Leahy

**Affiliations:** 1 Gastroenterology, West Hertfordshire Teaching Hospitals NHS Trust, London, GBR; 2 Pathology, West Hertfordshire Teaching Hospitals NHS Trust, London, GBR

**Keywords:** gastric emphysema, pneumatosis intestinalis, case report, chemotherapy, emphysematous gastritis

## Abstract

Emphysematous gastritis is a rare condition with a high mortality rate. We present a rare case of haemorrhagic emphysematous gastritis in a 70-year-old woman with a background of relapsed endometrioid ovarian cancer previously treated with chemotherapy and recent prednisolone use. A CT scan showed a grossly distended stomach with gas in the stomach wall and gas in the gastric and portal veins in the liver. The duodenum and small bowel were not dilated, suggesting gastric outlet obstruction potentially secondary to serosal deposits. Endoscopic evaluation showed an ischaemic oesophagus and posterior wall of the stomach, with necrosis of the greater curve. Histology showed complete loss of the gastric epithelium along with transmural necrosis along with intense acute and chronic inflammation. She was treated conservatively, as she was not fit for surgery due to her co-morbidities. She symptomatically improved and was discharged under the palliative care team. There are no current clear guidelines on treatment approaches. After a patient is haemodynamically stabilised, treatment options currently include surgical intervention (gastrectomy) or conservative options (fluid resuscitation, nasogastric decompression, broad-spectrum antibiotics/antifungals and supportive management). Historically, emphysematous gastritis was conventionally managed surgically. There has been a shift towards conservative management in recent literature, reporting good patient outcomes in patients successfully managed without surgical intervention.

## Introduction

Emphysematous gastritis is a rare condition with a reported mortality rate of 47.5-55% [1,2]. It is caused by the invasion of gas-producing organisms into the stomach wall due to damage to the gastric mucosa. With its high mortality rate, early detection and treatment are vital for improving patient outcomes and reducing the risk of complications. Gas in the stomach wall indicates a diagnosis of emphysematous gastritis or gastric emphysema, with the latter being a benign self-limiting process of non-infectious aetiology. These conditions must be differentiated to better guide treatment. We present a case of emphysematous gastritis in the context of multiple risk factors, including chemotherapy, recent steroid use and gastric outlet obstruction.

This article was previously presented and won a prize for "Best Poster - Gastroduodenal Category" at the 2024 BSG Live'24 British Society of Gastroenterology Conference on April 17-20, 2024 (DOI: 10.1136/gutjnl-2024-BSG.332).

## Case presentation

A 70-year-old woman with a background of relapsed endometrioid ovarian cancer and recent prednisolone use presented to the emergency department with syncope secondary to a two-month history of vomiting. She had no known cardiovascular risk factors such as peripheral vascular disease or coronary artery disease. She described intermittent, brown-coloured vomitus and epigastric pain worse on retching. She had a total abdominal hysterectomy with bilateral salpingo-oophorectomy and omentectomy alongside adjuvant platinum-based chemotherapy approximately a year before admission. She was due to restart chemotherapy in two weeks due to rising tumour markers. She was treated with domperidone and prednisolone in the community without improvement. On admission, her vital signs were: blood pressure of 87/64 mmHg, heart rate of 116 bpm, temperature 36.7 °C, respiratory rate of 18 rpm and oxygen saturation of 96% on room air. On examination, she had generalised abdominal tenderness, with no abdominal distension or signs of peritonism.

Investigations

**Figure 1:**
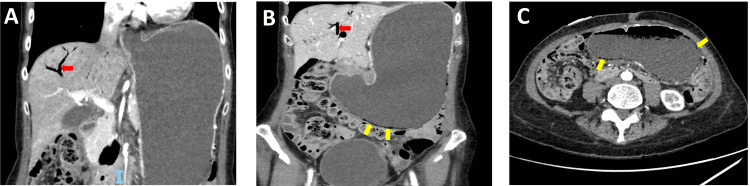
Coronal CT imaging demonstrating a grossly distended stomach with air in the portal venous system (red arrows in A and B); cross-sectional CT imaging showing air in the gastric wall (yellow arrows in B and C)

Biochemical evaluation on admission showed haemoglobin of 132 g/L, which dropped to 103 g/L with ongoing haematemesis, WCC 9.3 x10^9^/L, neutrophils 8.18 x10^9^/L, CRP 14.3 mg/L, urea 18 mmol/L, creatinine 68 µmol/L, potassium 2.8 mmol/L, prothrombin time 11.5 seconds, activated partial thromboplastin time 27.4 seconds and international normalised ratio 1. A blood smear showed left-shifted neutrophils, anisocytosis, poikilocytosis, elliptocytes and acanthocytes with rouleaux present. No causative organisms were isolated on blood cultures and gram stains did not demonstrate any organisms in the biopsy. Abdominal X-ray showed no dilated loops of bowel. She became confused and developed an oxygen requirement with acute shortness of breath. She also had witnessed episodes of coffee-ground vomiting. A computed tomography (CT) scan (Figure 1) showed acute pulmonary embolism, a grossly distended stomach with emphysematous gastritis and gas in the gastric vein and portal vein in the liver. It also showed a dilated, fluid-filled thoracic oesophagus. The duodenum and small bowel were not dilated, suggesting gastric outlet obstruction potentially secondary to suspicious serosal deposits.

**Figure 2:**
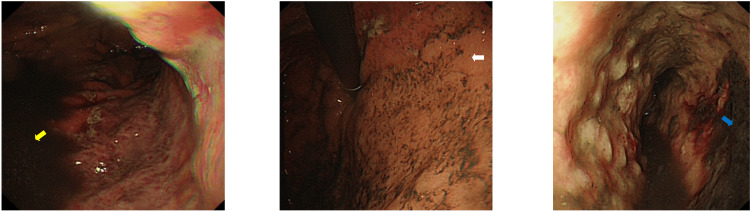
OGD images showing blood (yellow arrow); the entire posterior wall of the stomach appears ischaemic (white arrow) with necrotic black regions (blue arrow) on the greater curve OGD: oesophagogastroduodenoscopy

An oesophagogastroduodenoscopy (OGD) showed an ischaemic posterior wall of the stomach, with areas of necrosis on the greater curve. The oesophagus also appeared ischaemic, with no obvious gastric outflow obstruction (Figure 2).

**Figure 3:**
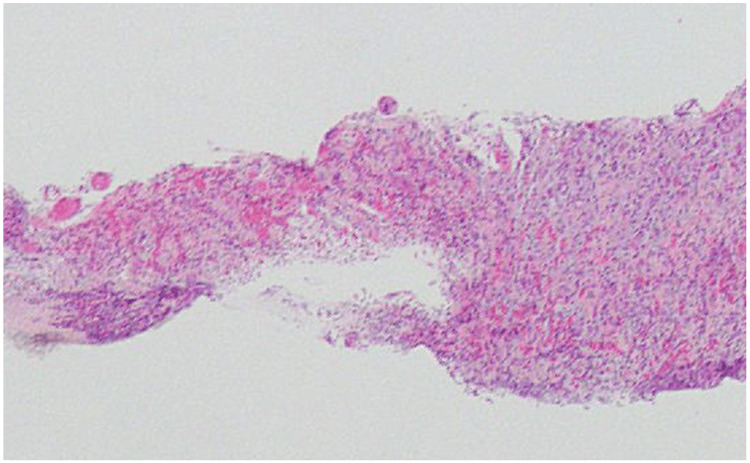
Ulcerated, necrotic, and intensely inflamed gastric mucosa with congestion and oedema H&E; magnification x100

Histology from a gastric biopsy during the OGD showed complete loss of the gastric epithelium along with transmural necrosis along with intense acute and chronic inflammation (Figure 3). The capillaries were congested, and the stroma was haemorrhagic and oedematous.

Treatment

The patient was first resuscitated to treat her shock, initially with crystalloids to address the septic component, then with blood products to treat the hypovolaemic component when her haemoglobin decreased in the context of ongoing bleeding. The patient was not suitable for surgery given her co-morbidities and low chance of surviving surgical intervention. As a result, she was treated conservatively. She was reported to be allergic to penicillin, so was treated with intravenous Teicoplanin and a dose of Gentamicin. A nasogastric (NG) tube was inserted for decompression, and the patient was started on high-dose omeprazole and sucralfate. A multidisciplinary team (MDT) approach was taken, with collaboration between gastroenterology, oncology, palliative care, dieticians and the patient. The outcome was that the patient was not suitable for further chemotherapy. There was a clinical conundrum with regards to her ongoing haematemesis and concurrent pulmonary embolism. Given her active bleeding being a contraindication to anticoagulation, alternatives were explored. She was not deemed suitable for an inferior vena cava (IVC) filter due to her frailty and poor oncological prognosis. Anticoagulation was held until her active bleeding had resolved. She was advised to withhold her anticoagulation and see a doctor if she had any episodes of haematemesis or melena. She was also reviewed by the dietician to help manage her nutritional needs. She was able to tolerate an oral liquid diet after antiemetics but was unable to tolerate a solid oral diet due to recurrent vomiting. She engaged well with physiotherapy and returned to her functional baseline. Palliative care input was vital for symptom control and community follow-up.

Outcome

She clinically improved, and her vomiting resolved. She was discharged approximately one month after admission. Her anticoagulation will be reviewed in three months. She was reviewed by the palliative care team during her admission, who would continue to follow her up in the community. She sadly died in the community six weeks later as a result of her advanced metastatic ovarian cancer.

## Discussion

The presence of gas in the intestinal mucosa (pneumatosis intestinalis) can be due to various aetiologies; however, gas in the stomach wall is a rarer form [3]. The stomach wall is usually resistant to infection due to its acidic environment and rich blood supply. Through various risk factors, a breakdown of these defences through a direct insult allows a gas-producing organism to invade. A case series of organisms isolated in patients with emphysematous gastritis found the common responsible organisms include Streptococci, E. coli, Enterobacter species, Clostridium welchii, Staphylococcus aureus, Pseudomonas aeruginosa, Klebsiella species and Candida species [4]. Proposed risk factors for emphysematous gastritis include malignancy, caustic ingestion, recent surgery, bowel obstruction, gastric distension, emesis, steroids, immunosuppressive medications, chemotherapy, alcohol and nonsteroidal anti-inflammatory drugs [5]. CT findings of gas in the stomach wall are present in both emphysematous gastritis and gastric emphysema. Emphysematous gastritis is due to the infective invasion of organisms into the wall and often presents with systemic signs of sepsis: hypotension, tachycardia, fever, abdominal pain as well as haematemesis/melaena. Gastric emphysema is a more benign process of non-infective aetiology where patients can often be haemodynamically stable and asymptomatic or experience mild abdominal pain. Gastric emphysema is a self-limiting process and can be managed conservatively, whereas emphysematous gastritis is associated with a high mortality rate, and early recognition and treatment initiation is key. They can be differentiated through history taking and clinical examination, with CT imaging being the modality of choice [6]. Emphysematous gastritis and gastric emphysema overlap in terms of radiographic characteristics; however, it has been reported that irregular mottled gas is a sign of emphysematous gastritis while linear streaks are characteristic of gastric emphysema [7]. An OGD can be useful and showed inflamed gastric mucosa with necrosis in over 50% of patients [1].

There are no current clear guidelines on treatment approaches. Treatment options currently include surgical intervention (gastrectomy) or conservative options (fluid resuscitation, nasogastric decompression, broad-spectrum antibiotics/antifungals and supportive management). Historically, emphysematous gastritis was conventionally managed surgically. There has been a shift towards conservative management in recent literature, reporting good patient outcomes in patients successfully managed without surgical intervention [6].

## Conclusions

In conclusion, we presented a case of emphysematous gastritis in a patient with multiple risk factors who was conservatively managed. Early recognition of this condition is important to initiate prompt treatment given its high mortality rate. Recognition is through a combination of clinical features, which can be non-specific, CT imaging and endoscopic evaluation, which will help differentiate between emphysematous gastritis and the more benign condition of gastric emphysema.
